# The molecular footprint of valproic acid in human hippocampal organoid

**DOI:** 10.1007/s12035-026-06260-6

**Published:** 2026-10-19

**Authors:** Bishwa R. Pokharel, Paul P. Cook, Shaw M. Akula

**Affiliations:** 1 Department of Microbiology & Immunology, Brody School of Medicine at East Carolina University, Greenville, NC 27834 USA; 2 Department of Internal Medicine, Brody School of Medicine at East Carolina University, Greenville, NC 27834 USA

**Keywords:** MiRNA, Hippocampus, Organoid, Autism, VPA

## Abstract

Autism spectrum disorder (ASD) is a multifaceted neurodevelopmental condition characterized by neuroinflammation, and disruption in neuronal development. Valproic acid (VPA) is a known environmental risk factor for ASD. Induced pluripotent stem cells (IPSCs IMR90-4)-derived human hippocampal organoids (hHOs) at 60 days of development were used to investigate VPA-induced changes in mRNA and microRNA (miRNA) profiles. The molecular effects of VPA on hHOs were deciphered by meaningfully integrating the ability of miRNA to regulate expression of their predicted mRNA targets crucial to altering neuroinflammatory pathways. Molecular footprint can be defined as the VPA-induced changes to the miRNA profile resulting in coordinated post-transcriptional (mRNA) regulation of neuronal and inflammatory pathways associated with ASD. Based on the results, the molecular footprint of VPA in hHOs was generated for the first time. These findings highlight the contribution of miRNA-mediated regulatory mRNA networks to VPA-induced neuroinflammation-associated with ASD in hHOs.

**Supplementary Information:**

The online version contains supplementary material available at https://doi.org/10.1007/s12035-026-06260-6.

## Introduction

Autism spectrum disorder (ASD) is a neurodevelopmental condition that affects individual’s communication, behavior, and social interaction [1]. Recent data from the CDC indicates that approximately 1 in 36 children in the United States and at least 78 million people worldwide is identified with ASD [1]. Globally, its prevalence is roughly 1 in 100 children, though rates vary significantly by region and diagnostic access [2]. Over the years, science has developed new therapies to prevent or treat various viral-and-bacterial infections, including immune related disorders. Unfortunately, we have yet to make any kind of breakthrough in treating or managing ASD partly because it is not a single disorder but a collection of closely related neurodevelopmental conditions [3]. Although genetic factors include a large portion of risk, they do not account for all cases. Many children with ASD do not carry known genetic variants associated with the disorder, which suggests that environmental exposures play an important role, especially during early development [4]. Maternal illness, air pollution, and metabolic disorders are among the prenatal and early-life exposures that have been linked to the risk of ASD. These factors may interfere with the development of the brain by causing oxidative stress, inflammation, or changes in gene regulation [5].

Valproic acid (VPA) is a mood-stabilizing and antiepileptic medication that easily passes through the placenta [6]. Numerous population-based studies have shown that children who are exposed to it during pregnancy are much more likely to acquire ASD and associated neurodevelopmental problems [7, 8]. Because this association is robust and reproducible, VPA has become one of the best-established environmental models of ASD in research.

The human brain has unique gene regulatory programs and developmental timing that cannot be completely captured in rodents [9]. Human brain organoids provide a way to study these processes directly. Brain organoids are three-dimensional (3D) tissues grown from human induced pluripotent stem cells (iPSCs) that contain neurons and glial cells organized in a manner similar to the developing brain [10]. Organoids can model early molecular events induced by environmental risk factors in a human-relevant system.

Hippocampus is a key brain region involved in learning, memory, and emotional regulation [11]. Neuroimaging and neuropathological studies show changes in hippocampal connectivity, synaptic structure, and gene expression in people with autism [12]. These observations suggest that abnormal hippocampal development may contribute to ASD symptoms. Therefore, we hypothesized that the human hippocampal organoids (hHOs) could serve as a good model to understand the VPA-induced changes to the molecular footprint crucial to developing ASD. The molecular footprint refers to the integrated regulatory network of miRNAs that act as post-transcriptional regulators of their target mRNAs capable of modulating key biological processes. In brain inflammation, this network modulates neuroimmune signaling and cellular responses through coordinated miRNA-mRNA interactions [13, 14]. Earlier studies have investigated changes to mRNA [15] and miRNA [16] profiles in brain organoids; however, they were not studied within the integrated framework. This study provides novel evidence that short-term VPA exposure induces coordinated changes to molecular footprint in hHOs critical to neuroinflammatory signals relevant to plausible ASD pathology.

## Methods

### Cells

Human embryonic kidney cells (HEK-293 T) were propagated in Dulbecco modified Eagle medium (DMEM) (Invitrogen, Carlsbad, CA) containing 10% charcoal-stripped fetal bovine serum, L-glutamine, and antibiotics as per earlier standard protocols [17].

### Human iPSCs was used to generate hHOs

Human iPSCs (IMR90-4, WiCell, p34, WB67847) derived from a female donor were cultured in a feeder-free environment on Matrigel coated plates as per standard protocols provided by WiCell Research Institute (Madison, WI). The single cell suspension of human iPSCs were induced to undergo neural differentiation and hippocampal organoid development as per earlier studies [18].

### Organoid fixation, frozen embedding, & sectioning

hHOs were fixed with 4% paraformaldehyde in 0.1 M sodium phosphate buffer (pH 7.4) at 4˚C overnight. The fixed hHOs were rinsed three times in PBS and placed in 30% Sucrose made in PBS (pH 7.2) and incubated overnight at 4˚C. These hHOs were embedded in optimal cutting temperature (OCT) compound in a disposable base mold, rapidly frozen with dry ice/2-methyl butane, and sectioned (10 microns thick) on a cryostat. The mounted sections were thawed on to charged slides which were stored in a closed slide box at −80˚C prior to performing immunofluorescence assay (IFA).

#### IFA

The slides with the sections of hHOs were thawed to room temperature for 15 min and washed 5 times in PBS gently to remove OCT. IFA of the sections were performed as per earlier studies [19]. Briefly, the sections were sequentially incubated with rabbit anti-PAX-6 (Cat # 60,433; Cell signaling technology, Danvers, MA), rabbit anti-nestin (Cat # ab92391, Abcam, Cambridge, UK), rabbit anti-OLIG2 (Cat # 65915S, Cell signaling technology), or rabbit anti-GFAP (Cat # 173,002, SYSY antibodies, Goettingen, Germany) antibodies; and anti-rabbit FITC (Sigma-Aldrich, Rockville, MD) prior to mounting using anti-fade reagent containing DAPI (Thermo Fisher Scientific, Waltham, MA). The stained slides were examined under a fluorescent Nikon microscope with a SPOT Advanced digital-imaging system (Diagnostic Instruments Inc.).

### High throughput mRNA and miRNA- sequencing

VPA (Selleck, Houston, TX) has been shown to have negligible effects on neuronal differentiation and minimal cytotoxicity at 1 mM [20]-a clinically relevant concentration [21]. hHOs at day 57 were provided with fresh organoid maturation medium [18], either supplemented with or without 1 mM VPA, for a duration of four days [22]. VPA was dissolved in the organoid maturation medium. After four days of VPA treatment, complete organoids (8–10) from each group were randomly chosen for total RNA extraction using the miRNeasy Mini Kit (Qiagen, 217,004). The RNA sample quality was assessed with the Agilent 2100 Bioanalyzer instrument. RNA samples exhibiting RNA integrity values over 8 were used for rRNA depletion RNA-seq library building. Both mRNA and miRNA-seq were performed by Genewiz (South Plainfield, NJ). The synthesized libraries were then used for RNA sequencing on the Illumina NovaSeq 6000 platform.

### RT-qPCR

RT-qPCR was performed to monitor mRNA levels as per our earlier studies [23]. RNA was extracted from the hHOs using RNAeasy mini kit (Qiagen, Germantown, MD). A 500 ng sample of RNA was incubated with 2 U of DNase I (ThermoFisher Scientific, Waltham, MA) and reverse transcribed in a solution containing 250 ng of random hexadeoxynucleotides and 50 U of Superscript RT (First-Strand cDNA Synthesis System for RT-PCR; ThermoFisher Scientific) in a final volume of 20 µl. A 2 µl volume of the synthesized cDNA was subjected to PCR reaction using specific forward and reverse primers (Supplementary Table 1). RNA was also used to monitor miRNA expression levels in the hHOs. For this purpose, total RNA were reverse transcribed using the All-in-oneTM miRNA RT-qPCR detection kit (GeneCopoeia, Rockville, MD, USA). The synthesized cDNA was directly used as the template for the PCR reaction in a Bio-Rad iQ5 Multicolour Real-Time PCR (Bio-Rad, Hercules, CA). In this system, MS2 RNA was used as an external reference for the quality of the extracted miRNAs, and RNU6B, RNU44, RNU48, and RNU49 were used for normalization. The expression levels of miRNAs were measured by employing a specific forward primer for the mature miRNA sequence, universal adaptor reverse primer (GeneCopoeia), and the SYBR green detection. The specific forward primers used to amplify different miRNAs are provided in Supplementary Table 2.

### Dual-luciferase reporter assay

Dual-luciferase reporter assay was conducted to appreciate the putative targets of the Novel miR-1052 (NmiR-1052) and NmiR-39. Four pairs of forward and reverse oligonucleotides (oligos) corresponding to the novel miRNA binding sites in the 3’-UTR (NDEL1, CRPPA, GPBP1, HS6ST2, and TGFBR3) and coding strand (CDS) (PTPRQ, TEK, and GABRB2) regions of mRNA targets were designed (Supplementary Table 3). All the oligos were synthesized (ThermoFisher Scientific, Waltham, MA) and cloned into pmirGLO dual-luciferase vector (Promega, Madison, WI, USA) to yield NDEL1/pmirGLO, GPBP1/pmirGLO, HS6ST2/pmirGLO, TGFBR3/pmirGLO, PTPRQ/pmirGLO, TEK/pmirGLO, CRPPA/pmirGLO, GABRB2/pmirGLO, respectively. HEK-293 T cells were plated onto 6-well plates. When the cells were 80% confluent, HEK-293 T cells were transfected with empty plasmid (pmirGLO plasmid), mRNA target/pmirGLO or co-transfected with target mRNA/pmirGLO, different concentrations of miRNA mimics (NmiR-39 and NmiR-1052 mimics), or 75 nM scrambled oligo controls (miR-SC) using FuGene HD (Promega, Madison, WI). At 24 h post transfection, the firefly and Renilla luciferase activity was measured using the dual luciferase reporter assay system (Promega) as per our earlier studies [24].

### Statistics

Student t test or One-way analysis of variance (ANOVA) was used appropriately to establish the statistical significance. ANOVA was performed using IBM SPSS v26 (Cary, NC) to determine significant differences between the treatment and control groups, followed by Tukey HSD post-hoc test for multiple comparisons. The ‘*p*’ value was established at the 0.05 level.

## Results

### Generation and validation of hHOs

**Fig. 1:**
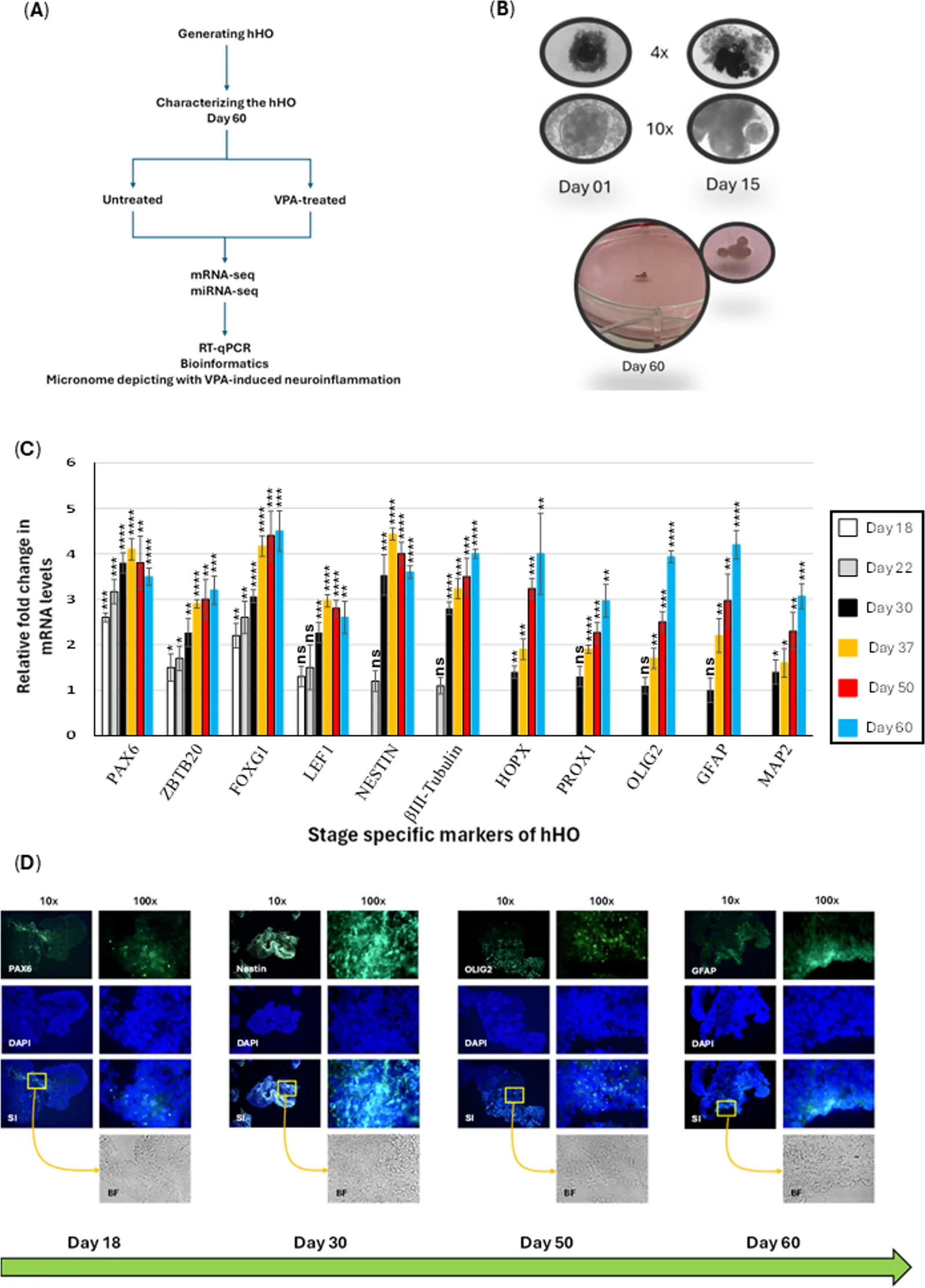
Generating hHOs. **A** A schematic depicting the workflow of this study. **B** Representative images of the generated hHOs. Microscopic images of hHOs at different magnifications on 01 and 15 days post inducing differentiation is provided. Actual images of a free floating hHO on day 60 is also shown (inset” magnified image of the hHO). **C** RT-qPCR data to monitor expression of various markers corresponding to development of hHOs on different days. Data are the mean ± SD (error bars) of four individual experiments. The fold change indicated is relative to the GAPDH expression that is considered as onefold. Student *t* test was performed to determine significant differences between the gene of interest and control reference gene (GAPDH). ‘****’, ‘***’, and ‘**’ denote *p* < 0.0001, *p* < 0.001, and *p* < 0.01, respectively. **D** IFA to monitor PAX6, nestin, OLIG2, and GFAP in hHOs at different time points during the development. Sections of hHOs were reacted with specific antibodies to PAX6, nestin, OLIG2, and GFAP followed by anti-rabbit-FITC antibodies. Stained sections were mounted using an anti-fade reagent containing DAPI (4,6-diamidino-2-phenylindole; Molecular Probes) and examined under a fluorescent Nikon microscope using respective filters. All the figures for different markers have representative images at 10 × mag and 100 × mag; including a corresponding brightfield image

A workflow summary of this study is presented in Fig. 1A. Human hippocampal organoids were generated from iPSCs using a differentiation protocol and kept in culture for up to 60 days (Fig. 1B). After embryoid body formation and early neural induction using dual SMAD inhibition, organoids were guided toward a dorsomedial telencephalic (DMT) identity by applying BMP4 for four days (day 10–14). This short BMP4 exposure helped promote dorsal forebrain patterning. Moreover, addition of Wnt3a after DMT induction promoted hippocampal primordium formation, while purmorphamine further improved regional identity through modulation of SHH signaling. During the maturation phase, organoids were maintained in differentiation medium supplemented with BDNF and GDNF to support neuronal survival and progressive maturation from day 30–60.

Neural induction of IMR90-4 iPSCs was confirmed by the expression of dorsal neural progenitor and developmental markers quantified by RT-qPCR (Fig. 1C). Early neuroectodermal commitment was supported by PAX6 expression, which increased from day 18 through day 37, followed by a modest decrease at day 60. DMT-stage markers, including ZBTB20, FOXG1, and LEF1, increased gradually during early-stage development of hHOs. FOXG1 and ZBTB20 expression gradually elevated from day 18 till day 60, whereas LEF1 expression increased from day 30 through day 60. Simultaneously, neural progenitor and neuronal differentiation markers showed distinct temporal patterns: NESTIN expression increased from day 30 till day 37 followed by a modest decrease, while βIII-Tubulin progressively increased from day 30 till day 60.

As the organoids developed toward day 60, hippocampal lineage- and maturation-associated markers became more pronounced (Fig. 1C). HOPX and PROX1 progressively increased from day 30 and day 37, respectively, to day 60, consistent with progressive hippocampal lineage specification. ZBTB20 also progressively increased during organoid development. PAX6 remained detectable at days 50 and 60. Markers associated with neuronal maturation and gliogenesis also increased during later stages of hHOs development, with OLIG2, GFAP, and MAP2 showing progressively higher expression at days 50 and 60. Notably, the continued increase in HOPX, PROX1, OLIG2, GFAP, MAP2, and βIII-Tubulin between days 50 and 60, together with the modest decrease in PAX6 and NESTIN, indicates continued developmental progression during this 10-day interval.

Immunostaining of selected marker proteins (PAX-6, Nestin, OLIG2, and GFAP) further demonstrated the development of a mature organoid, consistent with progressive DG development (Fig. 1D). The mRNA-seq analysis on the lab-developed hHOs on day 60 also revealed the total absence of the ventral telencephalic markers like NKX2-1 and GSX1, with only negligible expression of GSX2 and LHX6 (Supplementary Table 4). This data demonstrate that the organoids exhibited a pronounced bias towards dorsal telencephalic identity, hence validating effective hippocampal patterning with little ventral lineage contamination at this time.

The temporal expression patterns that were supplemented with Wnt3a and purmorphamine effectively directed organoids toward a hippocampal fate while limiting choroid plexus expansion as there were no visible cavity formation within the organoids. These results demonstrate the effective generation of transcriptionally verified and structurally stable hHOs that, by day 60, display staged development and DG-like characteristics.

### VPA-induced changes to the expression of genes regulating neuroinflammation

**Fig. 2:**
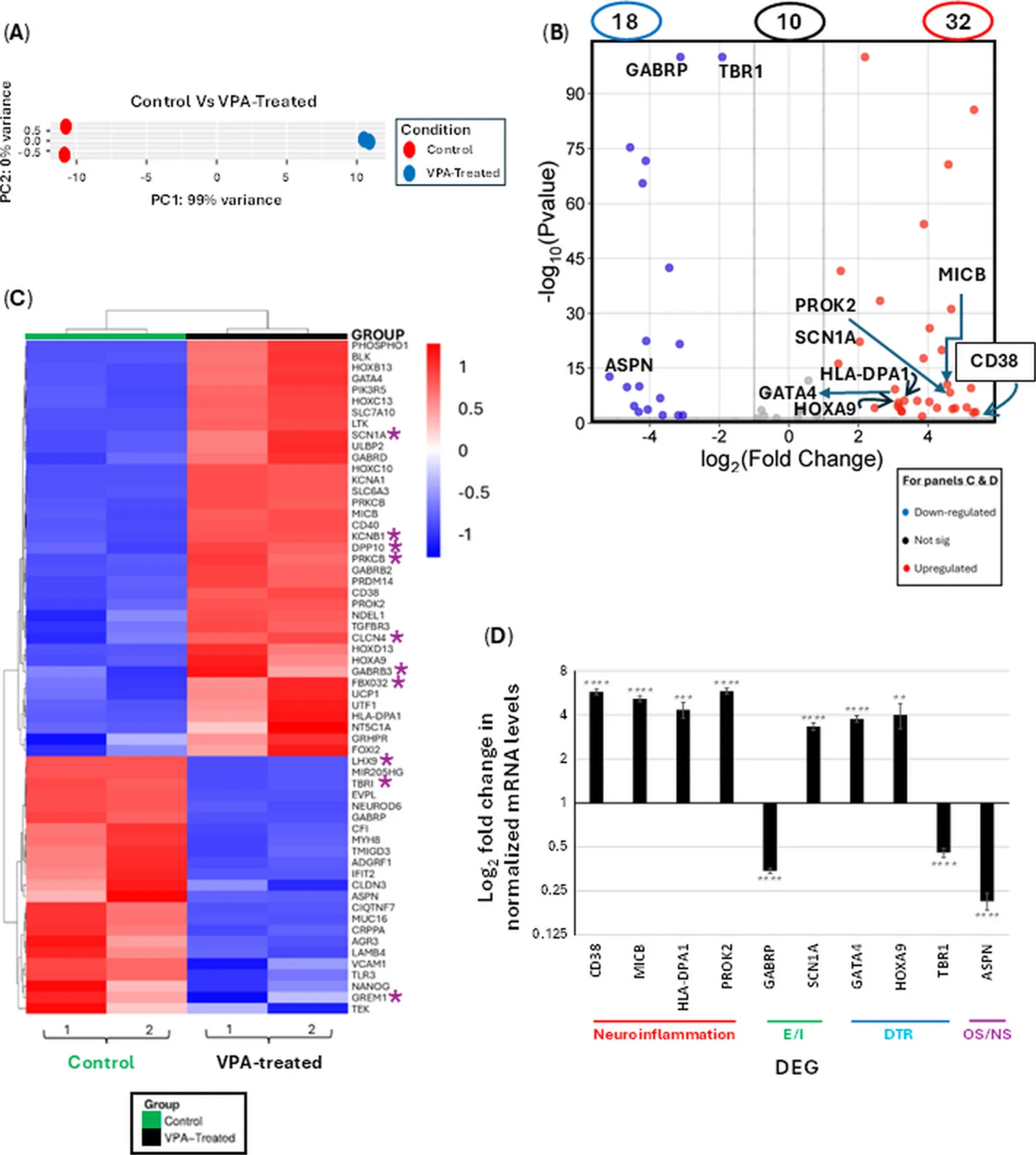
Heatmap of 60 DEGs showing hierarchical biclustering across experimental conditions. **A** PCA of mRNA-seq profiles from control hHOs and VPA-treated hHOs at day 60 revealed significant separation between the treatments. PC1 accounts for 99% of the total variation, effectively differentiating control samples, which cluster closely along the negative axis, from VPA-treated samples, which form a distinct cluster on the positive axis, indicating significant treatment-induced transcriptional divergence. PC2 is shown on the y-axis but accounts for low variation (0%), indicating limited intra-group variability and affirming that VPA exposure is the principal factor influencing global gene expression disparities in this dataset. **B** Volcano plot showing DEGs of select 60 genes related to neuroinflammation in VPA-treated hHOs compared to control untreated hHOs. Key: significantly upregulated genes (red), downregulated genes (blue), and non-significant genes (gray). **C** Heatmap displays normalized, log-transformed expression values for selected DEGs across untreated hHO controls and VPA-treated hHOs. Colors represent row-scaled expression levels (Z-scores), with red indicating higher expression and blue indicating lower expression relative to the gene mean. The data depicts values from duplicate set of experiments performed on two different days. Columns and rows were clustered using unsupervised hierarchical clustering, and the top annotation bar denotes sample group (control vs VPA-treated hHOs). Purple asterisk mark denotes dysregulated genes related to ASD and reported in earlier studies. **D** RT-qPCR data demonstrating fold change (log_2_ scale) in normalized mRNA levels of different genes in VPA-treated hHOs compared to untreated control hHOs is depicted. Bars represent average ± s.d. of three individual experiments. Student’s t test was performed to appreciate the statistical difference in VPA-treated hHOs compared to control hHOs. ‘****’, ‘***’, and ‘**’ denote *p* < 0.0001, *p* < 0.001, and *p* < 0.01, respectively

In order to determine VPA-induced transcriptomic landscape in 60-day hHOs, we employed mRNA-seq approach to evaluate the global genome expression profiles in 1 mM VPA-treated hHOs compared to untreated hHOs. High-throughput mRNA-seq of hHOs treated with VPA for 4 days and untreated hHO samples produced an average of 38.3 million paired-end reads per sample with an average of 94% unique mapped rate. Consistent with mRNA-seq data obtained from other eukaryotic cells [25], the sequencing reads mapped to annotated exons in human genome. Principal component analysis (PCA) was performed using the mRNA expression profiles to visualize the overall variation among samples and to assess whether the global transcriptomic profiles showed distinct clustering pattern upon VPA treatment. The two biological replicates within each experimental condition clustered closely together, indicating relatively low within-group variation, whereas the untreated control hHOs and VPA-treated hHOs formed clearly separated clusters (Fig. 2A). PC1 accounted for 99% of the total variance and distinctly separated the untreated control samples from the VPA-treated samples. In contrast, PC2 accounted for less than 1% of the total variance and reflected only a small degree of variation between biological replicates within each condition. Thus, the dominant source of transcriptomic variation in the dataset for any further analysis was associated with VPA treatment rather than variation between biological replicates. A graphical representation of the gene expression (MA plot) depicting fold change in logarithmic scale versus average of the normalized count of reads for every gene between the groups is provided (Supplementary Fig. 1A). Another common comparison method to visualize changes in expression values of differentially expressed genes between two groups is by generating volcano plots. Therefore, we generated two different volcano plots to depict differential expressions of the VPA-induced genes in hHOs: (i) a global volcano plot depicting the differential expression pattern of 23,043 genes in VPA-treated hHOs compared to untreated controls (Supplementary Fig. 1B); and (ii) a volcano plot depicting the differential expression pattern of 60 genes associated with neuroinflammation in VPA-treated hHOs compared to untreated hHOs (controls) (Fig. 2B).

Differentially expressed gene (DEG) analysis was conducted to compare the effects of VPA on hHOs compared to untreated hHOs. Heat map depicts the expression patterns of 60 dysregulated genes (Fig. 2C), underscoring the transcriptional effects of VPA exposure on hippocampal identity and function. These genes were ≥ twofold or ≤ 0.5 fold in VPA-treated hHOs compared to the untreated hHOs. VPA-exposure dysregulated 10 known genes that were confirmed by earlier studies to have a role in ASD and they were SCN1A, KCNB1, PRKCB, DPP10, CLCN4, GABRB3, FBXO32, LHX9, TBR1, and GREM1 [22, 26–28] (Fig. 2C). The normalized DEG data from mRNA-seq is provided as Supplementary Table 4. The DEGs can be categorized into 4 major groups based on their functional characteristics and their role in neuroinflammation: (**i**) chronic neuroinflammation and microglial activation; (**ii**) excitation/inhibition (E/I) imbalance and ASD-related; (**iii**) developmental transcriptional reprogramming (DTR); and (iv) oxidative stress (OS) & neurovascular stability (NS). The mRNA-seq data on the expression of 10 relevant genes was confirmed by performing RT-qPCR (Fig. 2D). In RT-qPCR, we tested only 10 out of 60 genes listed in the heatmap that were ≥ threefold or ≤ 0.5 fold in VPA-treated hHOs compared to the untreated hHOs. Asymmetric thresholds were used as practical criteria for selecting genes for RT-qPCR validation rather than as statistical definitions of differential expressions. A more stringent ≥ threefold cutoff was applied to prioritize genes showing robust expression changes and to reduce the inclusion of marginal candidates for RT-qPCR analysis. The ≤ 0.5-fold cutoff was retained for downregulated genes to identify transcripts showing at least a 50% reduction while maintaining representation of both expression directions. Such stringent fold-change criteria (≥ 3 or more-fold changes), to prioritize transcriptomic candidates for subsequent RT-qPCR validation has a precedence in human/mammalian studies [29–31].

As we confirmed the DEGs induced by VPA exposure to be significantly dysregulated via mRNA-Seq and RT-qPCR, we were curious to know whether these selected mRNAs were involved in coordinated disruption of major biological processes, instead of being isolated transcriptional events. Gene Ontology (GO) enrichment analysis clearly indicated a non-random merger of these genes on the biological pathways that mainly controlled inflammatory and immune response, neuron projection and K-channel, signifying that VPA exposure alters functional programs necessary for neuronal communication and network stability. Moreover, building up on axon, dendrites, and neuronal excitability changes indicate that the electrophysiological components of hippocampal circuitry are greatly influenced, indicating modified excitation changes rather than widespread cellular stress (Supplementary Figs. 2A, B). In addition, SynGO analysis revealed the instability of postsynaptic signaling and receptor localization mechanisms by revealing the overexpression of postsynaptic density structure, receptor diffusion trapping and synaptic cleft architecture. All these indicate that VPA-induced transcriptional alteration primarily focuses on synaptic structure and neurotransmission, rather than extensive metabolic networks (Supplementary Fig. 2C). The list of genes annotated in GO are submitted as separate supplementary tables (Supplementary Tables 5, 6, 7).

### VPA treatment induced changes to miRNA expression profiles

Small RNA sequencing of samples containing two untreated control hHOs and two VPA-treated hHOs yielded high-quality datasets for further differential expression analysis. Control samples generated 39,469,792 and 35,556,424 reads, respectively, while VPA-treated hHOs generated 42,098,759 and 44,541,239 reads. In all libraries, the average Phred quality-scores surpassed 36, with over 84% of bases attaining Q30 or above, indicating enhanced sequencing precision. Raw reads underwent processing using Illumina Bcl2fastq for base calling, followed by rigorous quality filtering using Trimmomatic (v0.30). Then, clean and high-quality reads were aligned to miRBase to identify the annotated (known) miRNAs and to the reference genome to predict novel miRNAs.

**Fig. 3:**
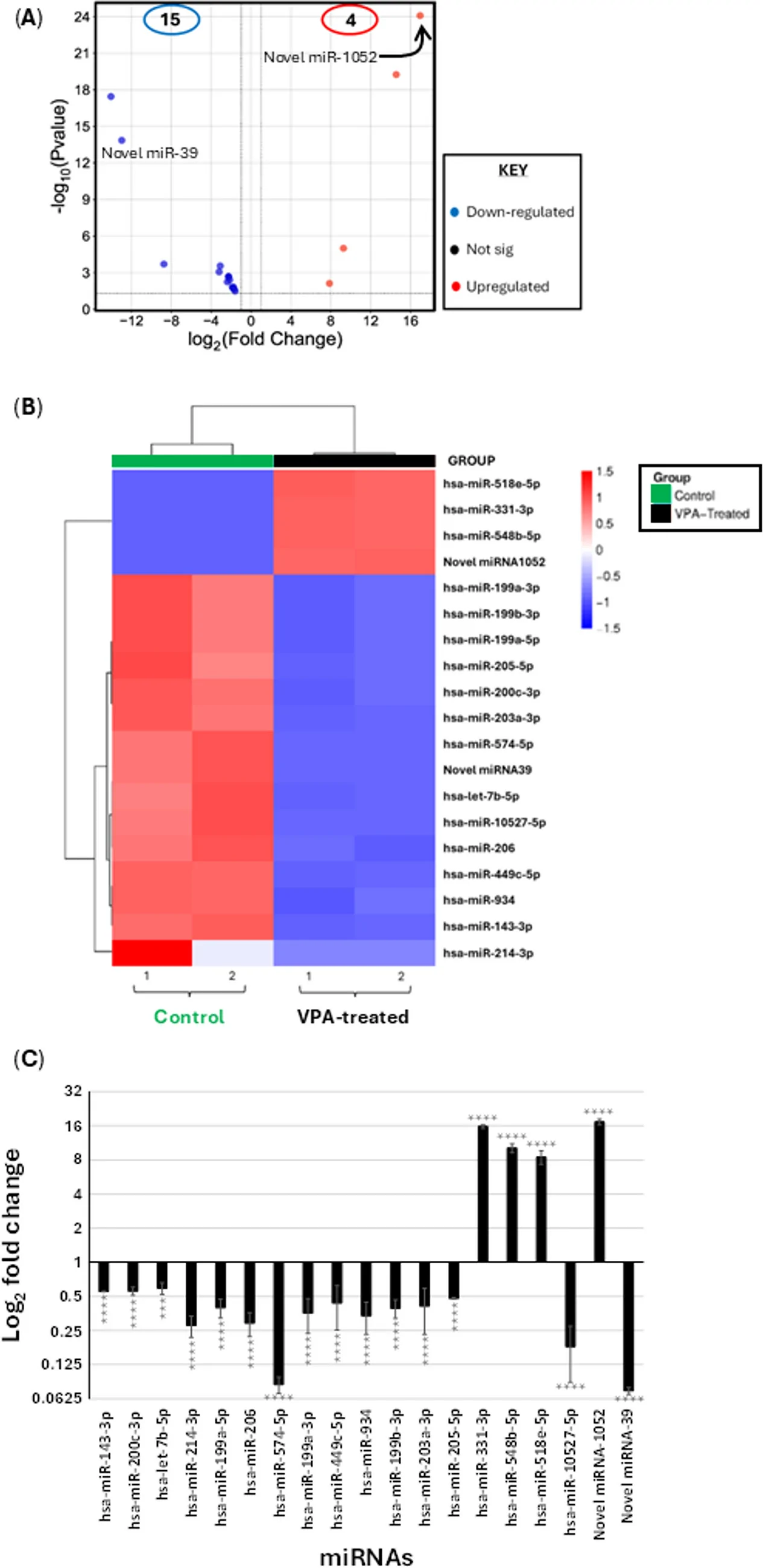
Differential expression of known and novel miRNAs in response to VPA. Volcano plots showing differential expression of (**A**) dysregulated 19 miRNAs with readout of 20 TPM or more at least in one treatment with adjusted p < 0.05 is depicted. **B** Heatmap displaying normalized miRNA-Seq readout for the above differentially expressed 19 miRNAs across control and VPA-treated hHOs; *p* < 0.05. **C** Expression of miRNAs detected by miRNA-seq was confirmed performing RT-qPCR. The RT-qPCR data was plotted for the 19 miRNAs that were differentially expressed in response to VPA treatment compared to untreated control hHOs. The relative expression of miRNAs was measured in terms of threshold cycle value (Ct) and normalized to snRNA RNU6B. The x-axis indicates the ID/name of the miRNAs and the y-axis indicates fold change in expression of these miRNAs in VPA-treated hHOs as compared to untreated hHOs (considered as onefold). Bars represent average ± s.d. of three individual experiments. Student’s t test was performed to appreciate the statistical difference between treatments. ‘****’, and ‘***’ denote *p* < 0.0001, and *p* < 0.001, respectively

Differentially expressed miRNAs in response to VPA-treatment of hHOs compared to untreated hHO controls were identified using volcano plot filtering (Fig. 3A). MiRNAs act both as a switch and as a fine-tuner of gene expression [32]. Fold changes to miRNA expression are small (∼1.5–twofold) [33] compared to those observed for genes. Such subtle changes to a group of miRNAs can collectively alter a signaling pathway resulting in a pronounced systemic effect [34]. Accordingly, we selected those miRNAs that were upregulated or downregulated by more than 1.5 fold (*p* value < 0.05) [35, 36]. Based on adjusted p < 0.5 value, log_2_ fold change ≥ or ≤ 1.6, and false detection rate (FDR) correction, we identified 17 known miRNAs and 2 novel miRNAs that were dysregulated (Fig. 3A). We focused on these 19 miRNAs as they were significantly altered with a median of ≥ 20 transcripts per million (TPM) in at least one of the groups with an adjusted p < 0.05. The clustering heat map showed that the expression levels of the 19 differentially differentiated known miRNAs that were significantly altered after VPA treatment (Fig. 3B). VPA treatment altered expression of miR-518e-5p, miR-331-3p, miR-548b-5p, miR-199a-3p, miR-199b-3p, miR-199a-5p, miR-205-5p, miR-200c-3p, miR-203a-3p, miR-574-5p, let-7b-5p, miR-10527-5p, miR-206, miR-449c-5p, miR-934, miR-143-3p, miR-214-3p, Novel miR-39, and Novel miR-1052. The miRNA-seq data was confirmed by performing RT-qPCR of selected miRNAs (Fig. 3C). The normalized DEG data from miRNA-seq is provided as Supplementary Table 8.

### The molecular footprint of VPA in hHOs

By sequencing miRNAs and mRNAs from the same hHOs, we had the unique opportunity to investigate the correlation between the two classes of RNAs (miRNAs and mRNAs) and their biological significance in the VPA-induced pathology in hHOs. Pearson’s correlation coefficient for all the possible miRNA-mRNA pairs in both the treatments based on the predicted miRNA targets was calculated. There was a weak inverse relationship (Pearson correlation coefficient *r* = −0.1086) observed between the miRNA and mRNA suggesting a decrease in one variable corresponding to an increase in the other.

Generation of the molecular footprint of VPA was a three-step process: (i) Identify mRNAs that were differentially expressed in response to VPA treatment. This was achieved by performing mRNA-seq (Fig. 2); (ii) Identify miRNAs that were differentially expressed in response to VPA treatment. This was achieved by performing miRNA-seq (Fig. 3); and (iii) to elucidate the downstream regulatory effects of the known miRNAs, putative target genes were identified using bioinformatic tools (Supplementary Table 9). The two novel miRNAs that seem to play a plausible role in VPA-induced inflammation are Novel miR-1052 (Supplementary Fig. 3A) and Novel miR-39 (Supplementary Fig. 3B); both are 22 bp long. The low energy secondary structures of the mature miRNAs are provided in Supplementary Figs. 3C, D. Bioinformatic tools were used to determine the putative targets of Novel miR-39 and Novel miR-1052. Details on the interactions between the novel miRNAs and their putative mRNA targets are provided in Supplementary Fig. 4. For these two novel miRNAs, the mRNA binding sites are canonical (in the 3’-UTR region) and non-canonical in nature (5’-UTR region and the coding strand) (Supplementary Fig. 4).

**Fig. 4:**
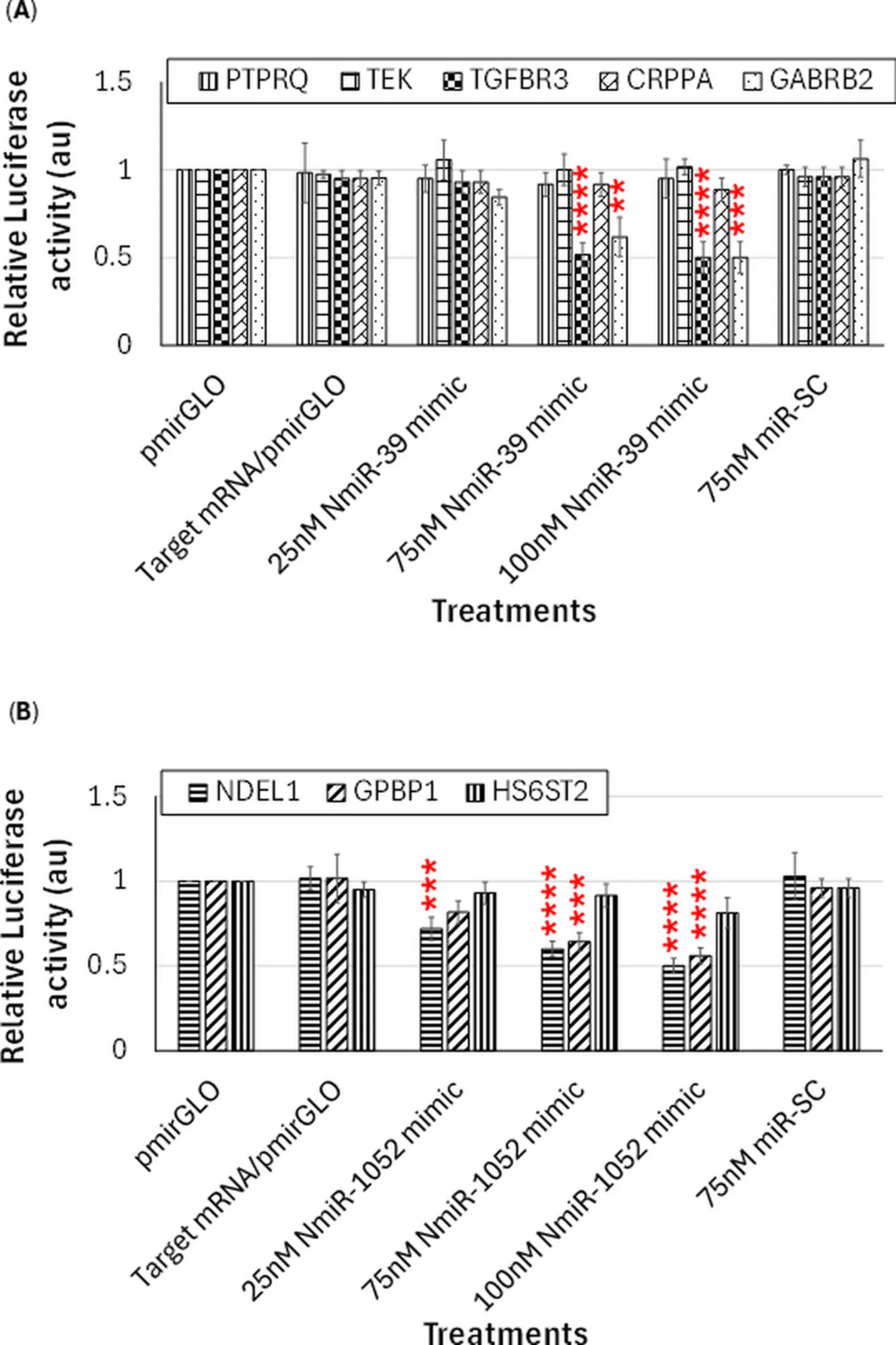
Luciferase reporter assay to establish putative mRNA targets of Novel (N) miR-39 and Novel miR-1052. HEK-293 T cells were (**A**) transfected with pmirGLO, pmirGLO expressing the 3’-UTR of TGFBR3 and CRPPA or CDS of PTPRQ, TEK, and GABRB2; co-transfected with the above plasmids and NmiR-39 mimic or scrambled controls (SC). **B** transfected with pmirGLO, pmirGLO expressing the 3’-UTR of NDEL1, GPBP1, and HSGST2; co-transfected with the above plasmids and NmiR-1052 mimic or scrambled controls (SC); Luciferase activity was monitored at 24 h post-transfection and was normalized against the effect of the miRNAs on the Firefly luciferase reporter. The relative luciferase activity for cells transfected with pmirGLO is considered as 1 au. The x-axis denotes the different treatments, and y-axis indicates the relative luciferase activity. One-way ANOVA was performed to compare between multiple groups. ‘**’, ‘***’, and ‘****’ denote *p* = 0.0065, *p* = 0.0010, *p* = 0.0005, and *p* < 0.0001, respectively

Bioinformatics predicted Novel miR-39 to bind and regulate expression of PTPRQ, TEK, TGFBR3, CRPPA, and GABRB2 (Supplementary Fig. 4). Bioinformatics predicted Novel miR-1052 to bind and regulate expression of NDEL1, GPBP1, and HS6ST2 (Supplementary Fig. 4). Luciferase reported assay was performed to confirm the bioinformatics-predicted targets for the above novel miRNAs. Transfection of HEK-293 T cells with Novel miR-39 mimic at 75 nM and 100 nM concentrations significantly lowered the luciferase activity of the TGFBR3/pmirGLO and GABRB2/pmirGLO vectors compared to PTPRQ/pmirGLO, TEK/pmirGLO, or CRPPA/pmirGLO vectors under the conditions tested (Fig. 4A). Transfection of HEK-293 T cells with Novel miR-1052 mimic significantly lowered the luciferase activity of NDEL1/pmirGLO vector at 25, 75, and 100 nM and the GPBP1/pmirGLO vector at 75 and 100 nM concentrations. However, Novel miR-1052 mimic did not alter the luciferase activity of HS6ST2/pmirGLO vector (Fig. 4B). The scrambled miRNA control (miR-SC) did not significantly alter the luciferase activity of any of the reporter plasmids tested. The results indicate that the putative targets for Novel miR-39 and Novel miR-1052 are TGFBR3, GABRB2 and NDEL1, GPBP1, respectively.

**Fig. 5:**
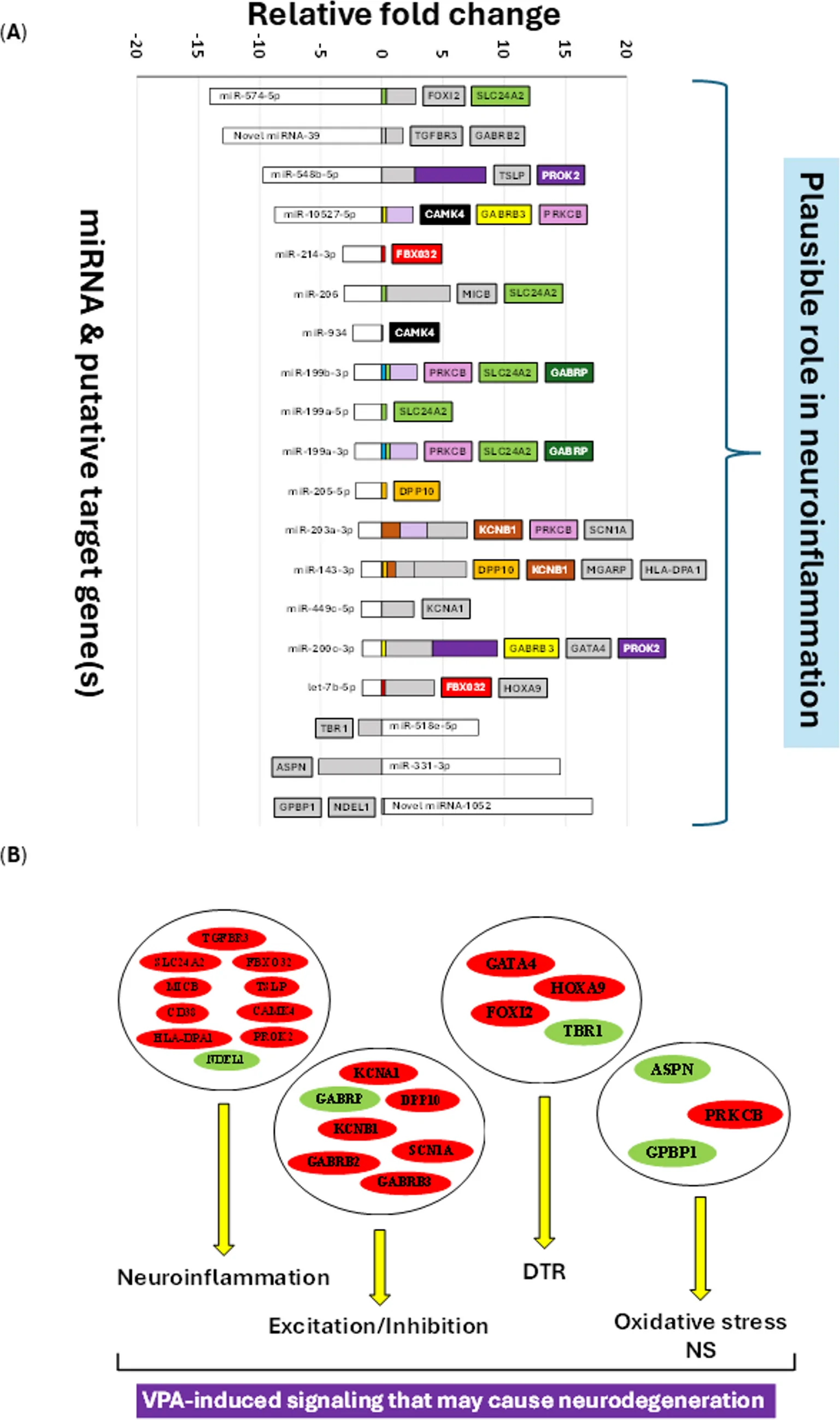
The molecular footprint of VPA in hHOs. **A** PART 1: Net effect of VPA treatment on the miRNA network. Bar graph shows the relative fold-change of known as well as candidate Novel miRNAs and their predicted target genes. Each miRNA is associated with its expected target(s), exhibiting inverse or coordinated expression patterns, in line with regulatory post-transcriptional interactions. **B** PART 2: Net effect of miRNA-induced changes to mRNA milieu on VPA-induced pathology. The effect of the miRNA-induced changes to the mRNA expression profile (panel A) on neuroinflammation, excitation/inhibition, DTR, and oxidative stress/neurovascular stability is depicted. Upregulated and down-regulated mRNAs are in red and green colors, respectively

Following these steps, we generated the molecular footprint representing the effects of VPA on hHOs. To keep it simple, the generated molecular footprint of VPA can be explained in two parts. Part 1 describes the net effect of VPA on miRNA regulatory network. In other words, part 1 describes the manner by which expression of 19 miRNAs that were altered in response to VPA treatment potentially regulate a plethora of predicted target mRNA expression. For example, a decrease in the expression of miR-548b-5p results in substantial increase in the expression of TSLP and PROK2 mRNA expression (Fig. 5A). Conversely, an increase in the expression of miR-331-3p results in a significant decrease in the ASPN mRNA expression (Fig. 5A). Part 2 describes the net effect of the putative miRNA targets’ (mRNA expression) milieu on neuroinflammation associated signaling, excitation/inhibition, DTR, and oxidative stress/neurovascular instability; all of which results in possible neurodegeneration (Fig. 5B).

## Discussion

Among many forms of neurodevelopmental disorders, ASD is one of the most common forms that affects young population [1]. Several studies have linked ASD to genetic alterations in response to environmental factors, rather than solely being associated to genetics [37, 38]. A substantial fraction of our understanding of the molecular neuro-pathophysiology of ASD is gained from rodent studies, especially with mice. These animal models are helpful entities, nevertheless, they are incompetent in entirely recapitulating human brain physiology and developmental progression. Moreover, mice scarcely characterize human-specific cortical growth, the existence of outer radial glia, developmental duration, or the wholesome complexities of neuronal maturation and synaptic connection. As a result, certain ASD-related signaling pathways display differences in rodents compared to the human fetal brain [39]. Because of these limitations, human brain organoid models derived from either embryonic or induced pluripotent stem cells have emerged as a powerful platform for exploring human brain development as well as neurodevelopmental disorders like ASD. There are several types of human brain organoids that are used to investigate brain pathologies including neuro-developmental disorders. Studies based on these brain organoids have identified crucial roles for several functional mRNAs in ASD [26], however, little is reported on the miRNAs that are dysregulated in ASD. Incidentally, miRNAs are upstream of mRNAs and have a key role in the biology of life.

VPA exposure is one of the most robust environmental models for studying ASD-like developmental disorder in CNS [40]. Our study investigated VPA-induced changes to the molecular footprint in hHOs (Figs. 1, 2). This was achieved by sequentially performing mRNA-seq (Fig. 2) and miRNA-seq (Fig. 3) on 60-day old hHOs that were treated with 1 mM VPA for a period of 4 days. ASD is a chronic neurodevelopmental condition [1]. However, we chose to study the molecular effects of a short 4 day VPA exposure because of the following reasons: (i) similar short VPA (0.5–1 mM) exposure times (3–5 days) have been used in earlier studies pertaining to forebrain and cortical organoids [22, 26]; and (ii) longer VPA exposure can produce substantial developmental toxicity, including reduced organoid growth, impaired neural progenitor cell (NPC) proliferation, and under some conditions, increased NPC apoptosis [41]. However, future studies incorporating different exposure durations and longitudinal analyses is critical to understanding the persistence and temporal evolution of the molecular alterations identified in this study. Based on the sequencing and RT-qPCR data, we constructed the molecular footprint of VPA-induced potential neuroinflammation (Fig. 5). The model included 16 miRNAs that were down regulated and 3 miRNAs that were upregulated in response to VPA-treatment of hHOs. Of these, two are novel miRNAs that are described by us for the first time. MiRNAs may regulate more than one gene expression [42]. Accordingly, these 19 miRNAs were predicted to target 24 genes involved in inflammation-associated signaling, E/I balance, transcriptional programming, oxidative stress, mitochondrial function, and ion homeostasis processes implicated in neuroinflammation and ASD.

Genes including SCN1A [43], KCNA1 [44], KCNB1 [45], and DPP10 [46] which were previously shown to regulate E/I balance were also altered in response to VPA treatment of hHOs (Fig. 5). It is well known that disruption of these pathways may alter neuron network and progress towards circuit malfunctions like in ASD. A group of genes associated with GABAergic signaling, including GABRB2 [47] and GABRB3 [48] which are critical for repressing synaptic transmission, were dysregulated in our model. Such alterations have been shown to affect signaling receptors seen in disorders like ASD [49]. Comparison with existing ASD and VPA-associated transcriptomic datasets provide additional context for the molecular alterations observed in our model. VPA exposure dysregulated 10 genes previously implicated in ASD: *SCN1A, KCNB1, PRKCB, DPP10, CLCN4, GABRB3, FBXO32, LHX9, TBR1,* and *GREM1* (Fig. 2C). Several of these genes show convergence with findings from other VPA-based neurodevelopmental models and human ASD-associated datasets [26, 41]. VPA treatment of human forebrain organoids altered expression of *CLCN4, DPP10, GABRB3, KCNB1, PRKCB,* and *SCN1A* and this was also observed in brains derived from ASD patients [22]. Interestingly, these genes are annotated in Simons Foundation Autism Research Initiative (SFARI) database. This convergence also extends to in vivo hippocampal models. Transcriptomic analysis of VPA-exposed prenatal rats identified extensive mRNA and miRNA dysregulation in the hippocampus, including altered regulatory networks associated with neurotransmitter uptake, long-term synaptic depression, and AMPA receptor function [50, 51]. Taken together, these observations indicate that the molecular alterations detected in our VPA-treated hHOs corroborate with earlier findings from human ASD-associated datasets, other VPA-treated in vivo and human brain organoid models. Nevertheless, direct equivalence should be interpreted cautiously because these studies differ in species, brain region, developmental stage, cellular composition, and VPA exposure paradigm. Thus, our findings support the ability of VPA-treated hHOs to reproduce a subset of ASD-associated molecular alterations within a hippocampal developmental context rather than recapitulating the complete molecular pathology of ASD.

Hippocampus is relevant to cognitive, memory, and behavioral processes affected in ASD [52]. However, ASD neuropathology is not restricted to hippocampus alone. Alterations involving the cerebral cortex, cerebellum, and other brain regions have also been implicated [53, 54]. Therefore, our model captured only a region-specific component of the broader neurodevelopmental pathology. Comparative studies using interconnected multi-region models may reveal molecular alterations that are not detectable in region specific hHOs.

Likewise, transcriptional regulators like TBR1, HOXA9, FOXI2, and GATA4 were differentially expressed in VPA-treated hHOs [27, 55–57]. TBR1 is especially noteworthy for its role in neurodevelopmental disorders and intellectual inability [58]. The abnormal expression of developmental transcriptomes implies that VPA exposure may interfere with neuronal differentiation and cortical patterning [26]. VPA-treatment also dysregulated genes related to neuroinflammatory signaling, like PROK2 [59], PRKCB [60], TSLP [61], TGFBR3 [62], and HLA-DPB1 [63]. Microglial activation and neuroinflammation are generally known as major factors in the pathogenesis of ASD [64]. Moreover, PRKCB is involved in intracellular signaling pathways that regulate inflammatory effects, while TGFBR3 regulates TGF-β signaling linked to immune control in CNS [22]. These findings suggest that VPA alters inflammation-associated gene expression that overlaps with molecular processes implicated in ASD. Importantly, the molecular alterations observed following VPA treatment in our study were not restricted to inflammation-associated pathways. Dysregulated genes were also associated with neuronal differentiation, E/I balance, ion homeostasis, and synaptic signaling, indicating that neuroinflammation-associated signaling represents one component of a broader neurodevelopmental response to VPA. Other processes implicated in ASD like, neural progenitor proliferation, cell-fate specification, neuronal migration, and synaptogenesis were not directly assessed in the present study and warrant investigation in future studies.

The combination of mRNA (Fig. 2) and miRNA-Seq (Fig. 3) data implies that various transcriptional changes identified in VPA-treated hHOs may be affected by miRNA-mediated post-transcriptional regulations. MiRNA modulates genes by binding to complementary nucleotide in target mRNAs, resulting in mRNA degradation or translational suppression [42]**.** Our study predicted miRNAs to regulate several of the above ASD-related genes. For example, members of the miR-199 gene family (miR-199a-3p, miR-199a-5p, and miR-199b-3p) regulate Ca^++^ signaling and synaptic activity via directly targeting expression of PRKCB, SLC24A2, and GABRP genes related to neuronal signaling and inflammatory pathways (Fig. 5) [22, 65]. Bioinformatic tools revealed miR-143-3p and miR-203a-3p to regulate HLA-DPB1, MGARP, CAMK4, KCNB1, DPP10 and CAMK4, KCNB1, PRKCB and SCN1A, respectively, that are involved in neural development and synaptic transmission. Among these, CAMK4, GABRB3, and KCNB1 also have regulatory effects on calcium-signaling, synaptic transmission, and neuronal excitability [48, 66, 67]. Differential expressions of these genes directly influence the E/I balance within synaptic circuits; a process linked to neuroinflammation and ASD (Fig. 5). Additionally, many miRNAs like miR-574-5p, miR-199a-3p, miR-199a-5p, miR-206 and miR-199b-3p may regulate expression of one gene, SLC24A2, which regulates neuronal Ca^++^ exchange and intracellular calcium homeostasis [22, 68]**.** Such findings of overlapping target amongst these miRNAs underpins the idea of multifaceted regulatory signaling, whereby numerous miRNAs come together to regulate same pathway or gene(s).

Apart from known miRNAs which were dysregulated in our dataset, our study also discovered two novel candidate miRNA, Novel miR-39 and Novel miR-1052, which are predicted to regulate various genes related to neuronal development and neuroinflammatory pathways. VPA-treatment down regulated expression of Novel miR-39 that resulted in dysregulation of TGFBR3 and GABRB2 (Fig. 5). VPA-treatment also enhanced expression of Novel miR-1052 (named as miR-12138 as per miRbase nomenclature) with a corresponding decrease in the expression of putative target gene (GPBP1 and NDEL1). NDEL1 controls intracellular transport, neuronal migration and microtubule organization, that are necessary for neuronal circuit formation [69, 70]. Disruption of such processes can interrupt synaptic connection, which has been associated in disorders including ASD. These genes are linked to oxidative stress, neuroinflammation, neuroimmune regulation, and E/I balance [71, 72].

Our study reports for the first time the VPA-induced molecular footprint in hHOs. The results from this study will serve as a conceptual framework to elucidate the molecular footprint model at a molecular level to better understand ASD. We strongly believe that our organoid model will serve as a platform to not only further our knowledge on the roles of miRNA-associated cell signaling in ASD but also other relevant neuroinflammatory processes. Future studies are aimed at validating the miRNA/mRNA interactions in sophisticated hHOs with microglia and vascular components to take it one step closer to being physiologically relevant.

### Limitations of this study

We are aware of the limitations of this study and they are as follows: (i) The hHOs used in this study were derived from a single female iPSCs. Therefore, potential sex-dependent differences in the molecular response to VPA could not be evaluated; (ii) The small number of biological replicates used for small RNA sequencing (n = 2 per group) reduces statistical power and may limit the detection of low-abundance miRNAs and the estimation of biological variability; and (iii) Microglia are major mediators of CNS immune responses [73, 74]. Brain vasculature contributes to cellular and functional complexity by providing oxygen and nutrients crucial to neural development and maturation [75, 76]. Absence of microglia and vascular components in our organoid model limits our ability to directly assess microglial activation and the full cellular complexity of neuroinflammation. Therefore, the inflammation-associated transcriptional and miRNA changes identified here should be interpreted as neuroinflammation-associated molecular signaling within the neural compartment rather than evidence of an established chronic neuroinflammatory state.

## Supplementary Information

Below is the link to the electronic supplementary material.

ESM 1 (PDF 561 KB)

ESM 2 (DOCX 18.2 KB)

ESM 3 (DOCX 15.7 KB)

ESM 4 (DOCX 16.0 KB)

ESM 5 (XLS 4.54 MB)

ESM 6 (TXT 60.1 KB)

ESM 7 (TXT 8.92 KB)

ESM 8 (TXT 1.60 KB)

ESM 9 (XLT 41.5 KB)

ESM 10 (TXT 77.8 MB)

## Data Availability

The data that support the findings of this study are available from the corresponding author upon reasonable request.
