# Supplementary material for: The molecular footprint of valproic acid in human hippocampal organoid
Source: Mol Neurobiol. 2026 Oct 19;63(1):979. doi: 10.1007/s12035-026-06260-6 (PMC13680533; doi:10.1007/s12035-026-06260-6)
Supplement: Supplementary file 1 [file 12035_2026_6260_MOESM1_ESM.pdf]

SUPPLEMENTARY DATA

The *micronome* footprint of valproic acid in human hippocampal organoid

Bishwa R. Pokharel[1], Paul P. Cook[2*] & Shaw M. Akula[1*]

Supplementary Figures: 1(A,B), 2(A-C), 3(A-D), 4 (**See below**)

Supplementary Tables:

Supplementary Table 1: Primer pairs for RT-qPCR (**Word**)
Supplementary Table 2: Forward primers for miRNA amplification (**Word**)
Supplementary Table 3: Oligos Used to Construct Luciferase Reporter Vectors (**Word**)
Supplementary Table 4: mRNA-seq data (**Excel**)
Supplementary Table 5, 6, 7: GO_Biological process (**Text**)
Supplementary Table 8: miRNA-seq data (**Excel**)
Supplementary Table 9: mRNA targets for known miRNAs (**Text**)

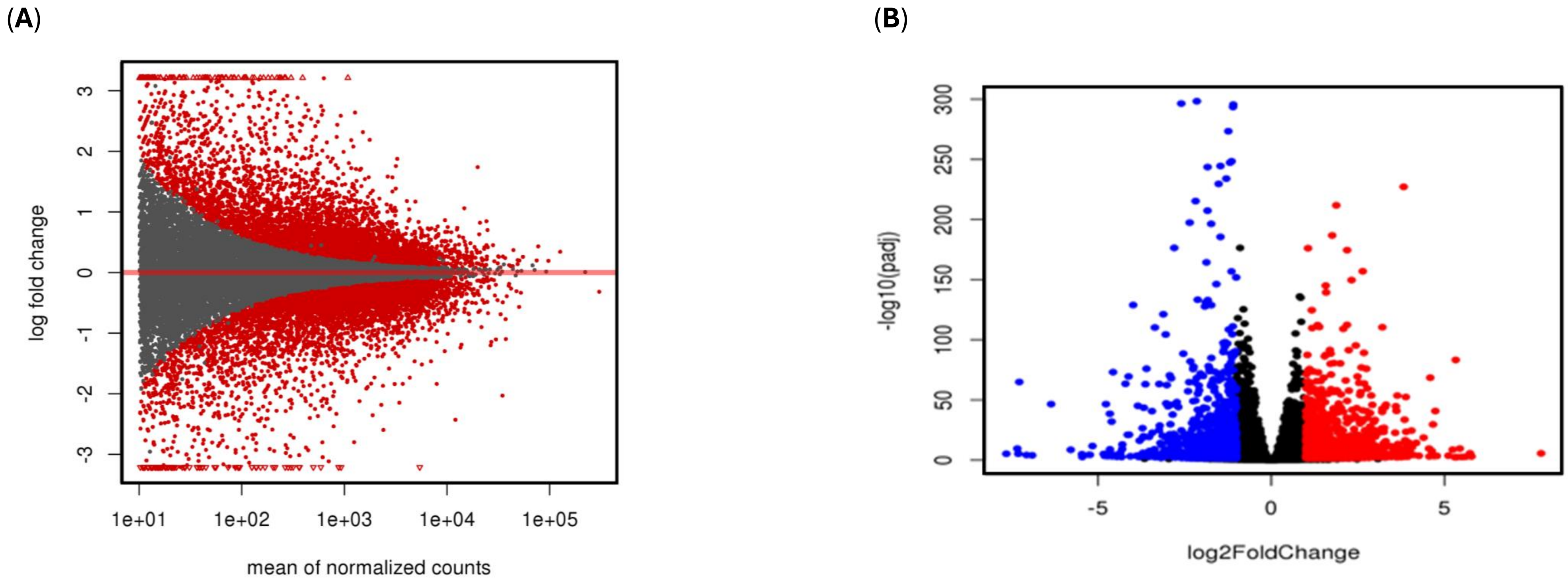

Supplementary Figure 1. (A) **MA plot showing differential gene expression between control and VPA-treated hHOs.** The MA plot displays $\log_2$ fold change (Control Vs VPA-treated) on the y-axis against the mean of normalized mRNA-seq counts on the x-axis (log scale). Each point represents an individual gene. Red dots indicate significantly differentially expressed genes (adjusted $p < 0.05$), whereas gray dots represent non-significant genes. The horizontal red line denotes zero $\log_2$ fold change, indicating no difference in expression between conditions. (**B**) **Global volcano plot showing differential expression of 23,043 genes between control and VPA-treated hHOs.** The volcano plot displays $\log_2$ fold change (control vs VPA-Treated) on the x-axis and $-\log_{10}$(adjusted p-value) on the y-axis. Each point represents an individual gene. Statistical significance was determined using Benjamini-Hochberg false discovery rate (FDR) correction, with significantly differentially expressed genes defined by adjusted $p < 0.05$.

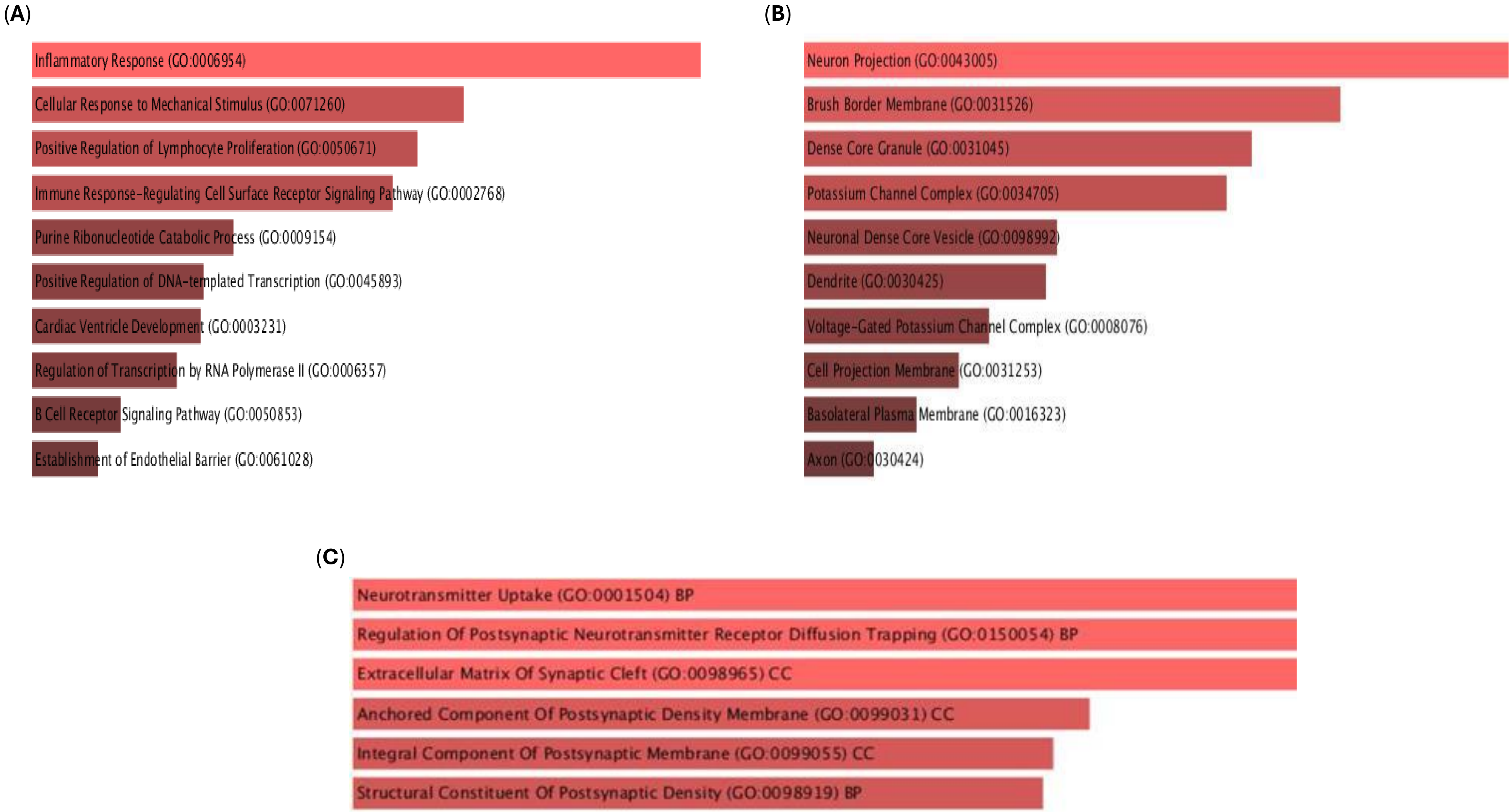

Supplementary Figure 2. GO of DEGs. GO of (A) biological process (B) cellular component and (C) SynGO enrichment analysis (via Enrichr) of 51 DEGs in hHO treated with 1mM of VPA. Bar length represents -log10 (enrichment p-value), with longer bars indicating stronger statistical enrichment.

(A) **Novel miRNA-39**

Mature miRNA: AAAAAACUGCAGUUACUUUUGU

Pre-miRNA:
aaaaguaauugcagguuuugcuauugaagguaauggaaaaaacugcaguuacuuuugu

(B) **Novel miRNA-1052**

Mature miRNA: CCCCCCACUGCUAAAUUUGACU

Pre-miRNA:
guaguggguaaucagaaguuacuaacauagugucauuaaaguugguauacaaccccccacugcuaaauuugacu

(C)

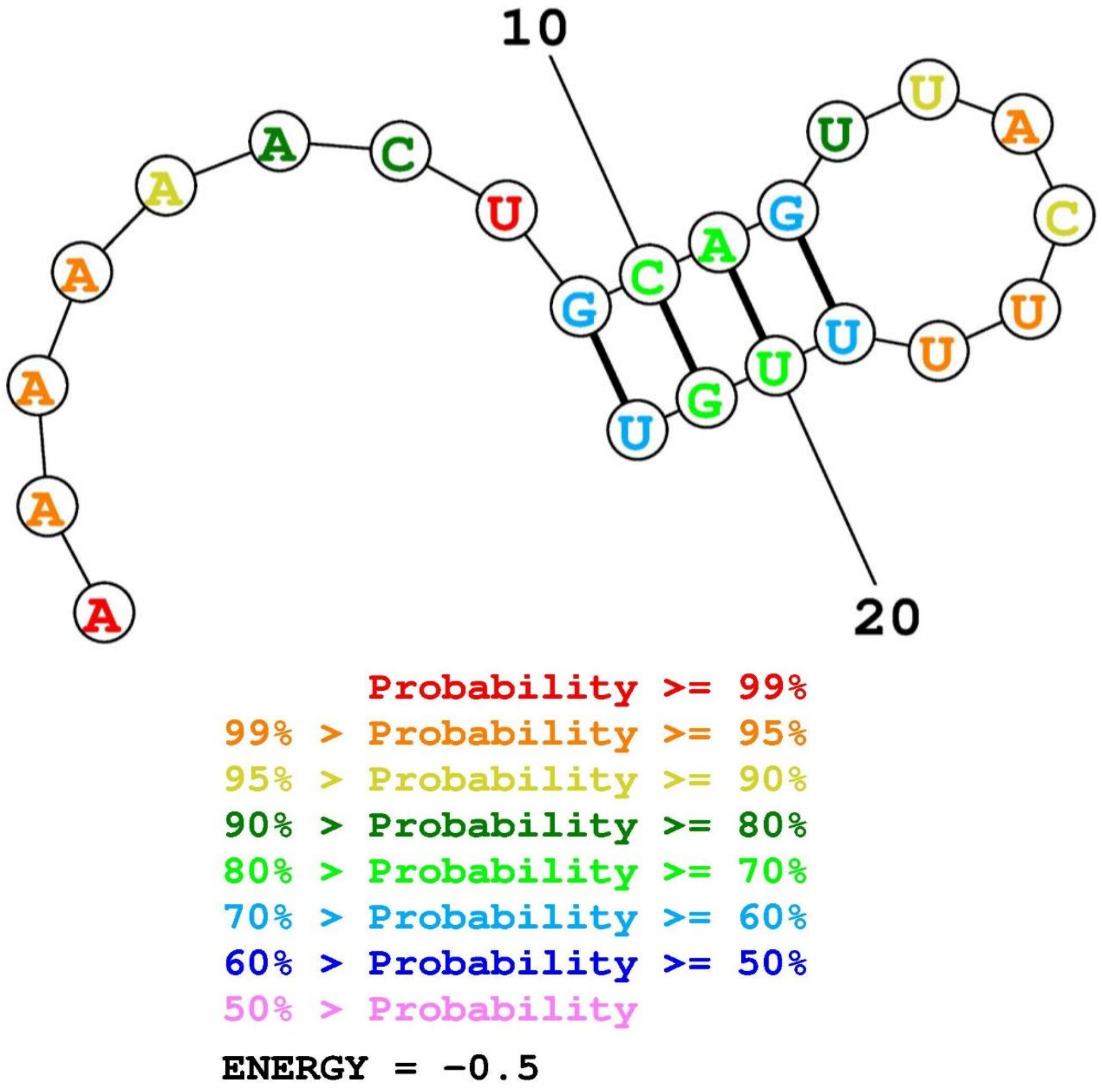

(D)

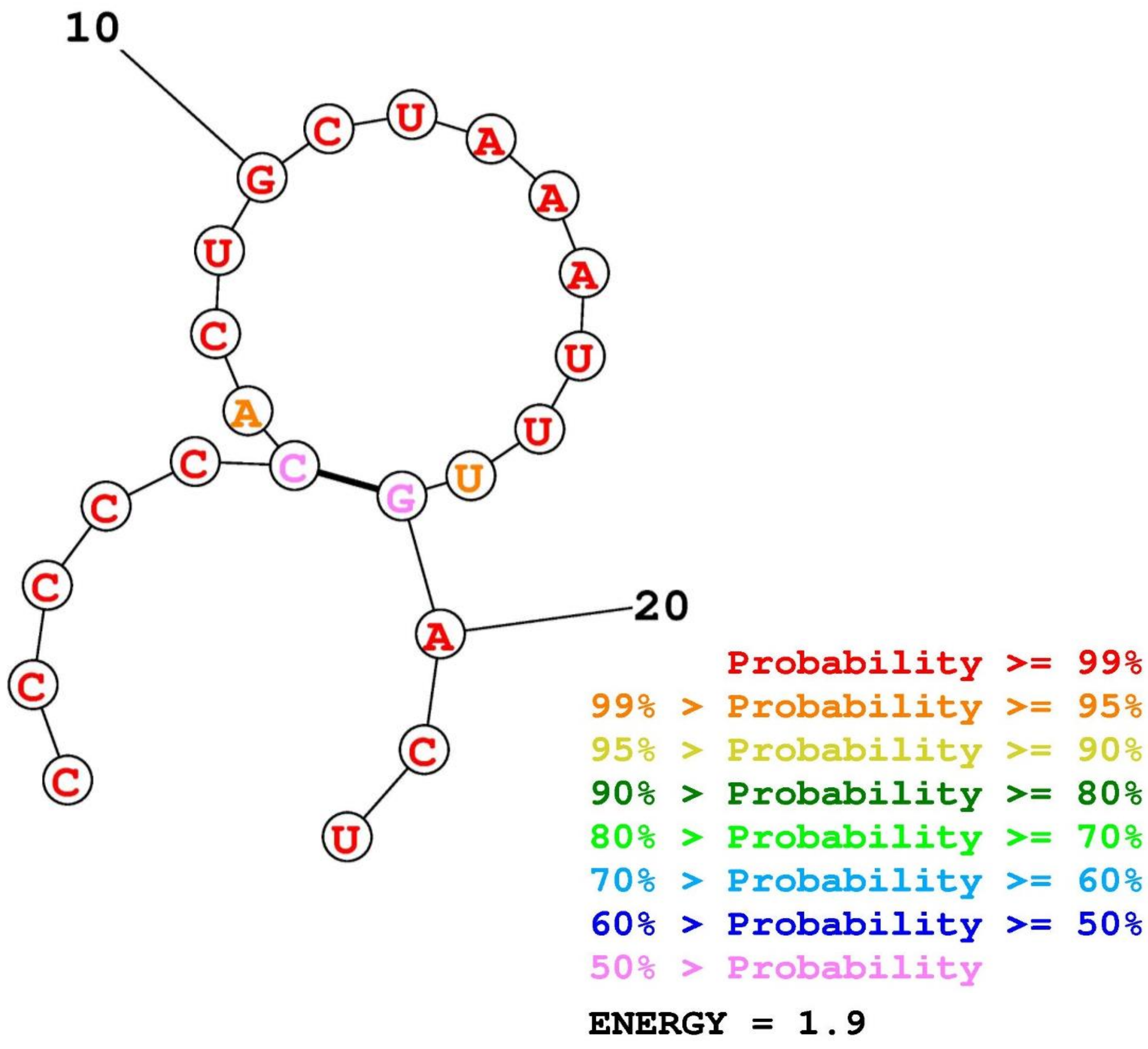

Supplementary Figure 3. Structure of mature Novel miRNA sequences. Sequence of the pre-miRNA and mature Novel miRNAs for (**A**) Novel miR-39 and (**B**) Novel miRNA-1052. The predicted low free energy secondary structure of (**C**) Novel miR-39 and (**D**) Novel miRNA-1052 is provided.

	miRNA / mRNA	ALIGNMENT	LOCATION [REGION]	Accession No.
Novel miRNA-1052	Novel miRNA-1052<br>NDEL1 3'-UTR	3'CCCCCCACTGCTAAATT**T**GACT5'<br>5'CCCCCCACTGCTAAATT**A**GACT3'	bp:61534-61555 [3'-UTR]	NG_029963.3
	Novel miRNA-1052<br>GPBP1 3'-UTR	3'CCCCCCA**C**T**G**CTAAATTTGACT5'<br>5'CCCCCCA**A**T**A**CTAAATTTGACT3'	bp:41500-41521 [5'-UTR]	NG_029503.2
	Novel miRNA-1052<br>HS6ST2 3'-UTR	3'CCCCCACTGCTAAATT5'<br>5'CCCCCACTGCTAAATT3'	bp:294876-294891 [5'-UTR]	NG_012840.2
Novel miRNA-39	Novel miRNA-39<br>PTPRQ*	3'AAAAACTGCAGTTACTTTTG5'<br>5'AAAAACTGCAGTTACTTTTG3'	bp:294876-294891 [Intronic miRNA within the CDS]	NG_012840.2
	Novel miRNA-39<br>TEK*	3'AAAAACTGCAGTTACTTTTG5'<br>5'AAAAACTGCAGTTACTTTTG3'	bp:10481-10500 [Intronic miRNA within the CDS]	NG_011828.2
	Novel miRNA-39<br>TGFBR3 3'-UTR	3'AAAAAAACTGCA**G**TTACTTTTGT5'<br>5'AAAAAAACTGCA**A**TTACTTTTGT3'	bp:89975-89996 [3'-UTR]	NG_027757.1
	Novel miRNA-39<br>CRPPA	3'AAAAACTGCA**G**TTACTTTTGT5'<br>5'AAAAACTGCA**A**TTACTTTTGT3'	bp:39121-39141 [3'-UTR]	NG_032690.2
	Novel miRNA-39<br>GABRB2*	3'AAAAACTGCA**G**TTACTTTTGT5'<br>5'AAAAACTGCA**A**TTACTTTTGT3'	bp:198261-198261 [Intronic miRNA within the CDS]	NG_047050.1

Supplementary Figure 4

KEY: Bold letters in black and red denotes non-complementary base(s): * denotes non-canonical binding
