# Supplementary material for: The molecular footprint of valproic acid in human hippocampal organoid
Source: Mol Neurobiol. 2026 Oct 19;63(1):979. doi: 10.1007/s12035-026-06260-6 (PMC13680533; doi:10.1007/s12035-026-06260-6)
Supplement: Supplementary file 2 [file 12035_2026_6260_MOESM2_ESM.docx]

**Supplementary TABLE 1. Primer pairs for RT-qPCR.**

| **S.No.** | **Genes** | **Primer Sequence (5' -> 3')** | | **Tm (°C)** |
| --- | --- | --- | --- | --- |
| 1 | CD38 | Forward | AGACTGCCAAAGTGTATGGGA | 60.8 |
|  |  | Reverse | GCAAGGTACGGTCTGAGTTCC | 62.4 |
| 2 | HLA-DPA1 | Forward | ATGCGCCCTGAAGACAGAATG | 62.8 |
|  |  | Reverse | ACACATGGTCCGCCTTGATG | 62.5 |
| 3 | PROK2 | Forward | GTGACAAGGACTCCCAATGTG | 60.6 |
|  |  | Reverse | TGACCCAGATACTGACAGCAC | 61.2 |
| 4 | SCN1A | Forward | TCTCTTGCGGCTATTGAAAGAC | 60.4 |
|  |  | Reverse | GGGCCATTTTCGTCGTCATCT | 62.7 |
| 5 | MICB | Forward | TGGAGACTCAAGAATCGACAGT | 60.5 |
|  |  | Reverse | CTGCATAGCGCGATAGTGTG | 61.0 |
| 6 | GATA4 | Forward | CGACACCCCAATCTCGATATG | 60.0 |
|  |  | Reverse | GTTGCACAGATAGTGACCCGT | 62.1 |
| 7 | HOXA9 | Forward | TACGTGGACTCGTTCCTGCT | 62.7 |
|  |  | Reverse | CGTCGCCTTGGACTGGAAG | 60.0 |
| 8 | TBR1 | Forward | GCAGCAGCTACCCACATTCA | 62.5 |
|  |  | Reverse | AGGTTGTCAGTGGTCGAGATA | 61.4 |
| 9 | GABRP | Forward | CTTGGCCTTCGTGTGTCTGAG | 62.9 |
|  |  | Reverse | CCGACCTCGACGTTGAACT | 61.6 |
| 10 | ASPN | Forward | CTCTGCCAAACCCTTCTTTAGC | 61.1 |
|  |  | Reverse | CGTGAATAGCACTGACATCCAA | 60.4 |
| 11 | PAX6 | Forward | TGGGCAGGTATTACGAGACTG | 61.0 |
|  |  | Reverse | ACTCCCGCTTATACTGGGCTA | 61.9 |
| 12 | ZBTB20 | Forward | GACAGGATCTACTCGGCACTC | 61.4 |
|  |  | Reverse | ACTGCGCCGCTGTAAAAAGA | 62.7 |
| 13 | FOXG1 | Forward | GAGCGACGACGTGTTCATC | 60.9 |
|  |  | Reverse | GCCGTTGTAACTCAAAGTGCTG | 62.2 |
| 14 | LEF1 | Forward | AGAACACCCCGATGACGGA | 62.6 |
|  |  | Reverse | GGCATCATTATGTACCCGGAAT | 60.0 |
| 15 | Nestin | Forward | GCTCGTCATTTGCGCGAATAC | 62.6 |
|  |  | Reverse | TTTCTGTACGCAGCGATTTCTAT | 60.1 |
| 16 | βIII-Tubulin | Forward | GGCCAAGGGTCACTACACG | 62.3 |
|  |  | Reverse | GCAGTCGCAGTTTTCACACTC | 62.0 |
| 17 | HOPX | Forward | GAGACCCAGGGTAGTGATTTGA | 60.8 |
|  |  | Reverse | AAAAGTAATCGAAAGCCAAGCAC | 60.0 |
| 18 | PROX1 | Forward | AAAGGACGGTAGGGACAGCAT | 63.0 |
|  |  | Reverse | CCTTGGGGATTCATGGCACTAA | 62.0 |
| 19 | OLIG2 | Forward | CCAGAGCCCGATGACCTTTTT | 62.4 |
|  |  | Reverse | CACTGCCTCCTAGCTTGTCC | 62.0 |
| 20 | GFAP | Forward | CTGCGGCTCGATCAACTCA | 62.1 |
|  |  | Reverse | TCCAGCGACTCAATCTTCCTC | 61.3 |
| 21 | MAP2 | Forward | CTCAGCACCGCTAACAGAGG | 62.3 |
|  |  | Reverse | CATTGGCGCTTCGGACAAG | 61.8 |
| 22 | GAPDH | Forward | GGAGCGAGATCCCTCCAAAAT | 61.6 |
|  |  | Reverse | GGCTGTTGTCATACTTCTCATGG | 60.9 |
