# Supplementary material for: The molecular footprint of valproic acid in human hippocampal organoid
Source: Mol Neurobiol. 2026 Oct 19;63(1):979. doi: 10.1007/s12035-026-06260-6 (PMC13680533; doi:10.1007/s12035-026-06260-6)
Supplement: Supplementary file 3 [file 12035_2026_6260_MOESM3_ESM.docx]

**Supplementary Table 2: Forward primers for miRNA amplification.**

| **S.No.** | **miRNAs** | **Forward Primer (5' -> 3')** | **Tm (°C)** |
| --- | --- | --- | --- |
| 1 | hsa-miR-574-5p | GTGAGTGTGTGTGTGTGAGTGTGTAAAAA | 58.7 |
| 2 | hsa-miR-206 | GTGGAATGTAAGGAAGTGTGTGGAAAAAA | 57.3 |
| 3 | hsa-miR-214-3p | ACAGCAGGCACAGACAGGCAGT | 58.6 |
| 4 | hsa-miR-199b-3p | GGACAGTAGTCTGCACATTGGTTAAAAA | 57 |
| 5 | hsa-miR-199a-3p | GGACAGTAGTCTGCACATTGGTTAAAAAA | 57.3 |
| 6 | hsa-miR-199a-5p | CCCAGTGTTCAGACTACCTGTTC | 57.1 |
| 7 | hsa-miR-205-5p | TCCTTCATTCCACCGGAGTCTGAAA | 57.7 |
| 8 | hsa-miR-934 | GTGTCTACTACTGGAGACACTGGAAAAA | 58.5 |
| 9 | hsa-miR-143-3p | GGTGAGATGAAGCACTGTAGCTCAAAAA | 58.5 |
| 10 | hsa-miR-203a-3p | GGGTGAAATGTTTAGGACCACTAGAAAAA | 57.3 |
| 11 | hsa-miR-449c-5p | TAGGCAGTGTATTGCTAGCGGCTGT | 59.3 |
| 12 | hsa-miR-200c-3p | GTAATACTGCCGGGTAATGATGGAAAAA | 57 |
| 13 | hsa-let-7b-5p | GTGAGGTAGTAGGTTGTGTGGTTAAAAA | 57 |
| 14 | NovelmiRNA-1052 | GCCCCCCACTGCTAAATTTGACTAAAAA | 58.5 |
| 15 | NovelmiRNA-39 | GGGAAAAAACTGCAGTTACTTTTGTAAAAAA | 55.1 |
| 16 | hsa-miR-331-3p | GCCCCTGGGCCTATCCTAGAA | 58.3 |
| 17 | hsa-miR-548b-5p | GGGAAAAGTAATTGTGGTTTTGGCCAAAAAA | 57.8 |
| 18 | hsa-miR-518e-5p | CTCTAGAGGGAAGCGCTTTCTG | 56.7 |
| 19 | hsa-miR-10527-5p | AAAGCAAATGTTGGGTGAACGGCAAAA | 56.7 |
