# Supplementary material for: The molecular footprint of valproic acid in human hippocampal organoid
Source: Mol Neurobiol. 2026 Oct 19;63(1):979. doi: 10.1007/s12035-026-06260-6 (PMC13680533; doi:10.1007/s12035-026-06260-6)
Supplement: Supplementary file 4 [file 12035_2026_6260_MOESM4_ESM.docx]

**Supplementary TABLE 3**. Oligos Used to Construct Luciferase Reporter Vectors.

| Target Gene | Oligos (5’-3’) |
| --- | --- |
| NDEL1 | **NDEL1.F**  AAACTAGCGGCCGCTAGTGAGTGCCTAGCCTCCAGGTGGGGGCTCCTT  **NDEL1.R**  CTAGAAGGAGCCCCCACCTGGAGGCTAGGCACTCACTAGCGGCCGCTAGTTT |
| GPBP1 | **GPBP1.F**  AAACTAGCGGCCGCTAGTTGGGACTGAGACTGGTTGTGGGGGAGGGAT  **GPBP1.R**  CTAGATCCCTCCCCCACAACCAGTCTCAGTCCCAACTAGCGGCCGCTAGTTT |
| HS6ST2 | **HS6ST2.F**  AAACTAGCGGCCGCTAGTAGTACCCTGAGTTGCCTGAAGGGGGGAT  **HS6ST2.R**  CTAGATCCCCCCTTCAGGCAACTCAGGGTACTACTAGCGGCCGCTAGTTT |
| PTPRQ | **PTPRQ.F**  AAACTAGCGGCCGCTAGTGGAGTTGGAAGAACTGGAGTTTTTATTGCT  **PTPRQ.R**  CTAGAGCAATAAAAACTCCAGTTCTTCCAACTCCACTAGCGGCCGCTAGTTT |
| TEK | **TEK.F**  AAACTAGCGGCCGCTAGTATGAAGCATGCCACCCTGGTTTTTACGGGT  **TEK.R**  CTAGACCCGTAAAAACCAGGGTGGCATGCTTCATACTAGCGGCCGCTAGTTT |
| TGFBR3 | **TGFBR3.F**  AAACTAGCGGCCGCTAGTAATGTGAAACACATGCCAGTTTTTAAAATT  **TGFBR3.R**  CTAGAATTTTAAAAACTGGCATGTGTTTCACATTACTAGCGGCCGCTAGTTT |
| CRPPA | **CRPPA.F**  AAACTAGCGGCCGCTAGTTATTTTAAAGGGTCTCTAGTTTTTCTGAAT  **CRPPA.R**  CTAGATTCAGAAAAACTAGAGACCCTTTAAAATAACTAGCGGCCGCTAGTTT |
| GABRB2 | **GABRB2.F**  AAACTAGCGGCCGCTAGTCTTATCACCAAGAAGGTTGTTTTTTCCACT  **GABRB2.R**  CTAGAGTGGAAAAAACAACCTTCTTGGTGATAAGACTAGCGGCCGCTAGTTT |

* miRNAs binding site is highlighted in RED.
